# Formononetin: a review of its source, pharmacology, drug combination, toxicity, derivatives, and drug delivery systems

**DOI:** 10.3389/fphar.2025.1534798

**Published:** 2025-03-03

**Authors:** Min Jin, Linfang Wei, Jianhua Wang, Yuehong Shen, Lei Gao, Fan Zhao, Qianying Gao, Yifei Ma, Yongyan Sun, Ying Lin, Guanjie Ji, Pingping Cai, Rugen Yan

**Affiliations:** ^1^ College of Pharmacy, Shandong University of Traditional Chinese Medicine, Jinan, China; ^2^ Affiliated Hospital of Integrated Traditional Chinese and Western Medicine, Nanjing University of Chinese Medicine, Nanjing, China; ^3^ Department of Thyroid and Breast Surgery, Affiliated Hospital of Integrated Traditional Chinese and Western Medicine, Nanjing University of Chinese Medicine, Nanjing, China; ^4^ School of Integrated Chinese and Western Medicine, Nanjing University of Chinese Medicine, Nanjing, China; ^5^ Weifang Hospital of Traditional Chinese Medicine, Weifang, China; ^6^ Department of Traditional Chinese Medicine, Shandong Provincial Hospital Affiliated to Shandong First Medical University, Jinan, China

**Keywords:** formononetin, pharmacology, drug combination, toxicity, derivatives, drug delivery systems

## Abstract

Formononetin (FMN) is a common natural metabolite that can be extracted and isolated from some common botanical drugs. In recent years, FMN has garnered increasing attention due to its beneficial biological activities. In this paper, we systematically summarize the sources of FMN and provide a comprehensive review of its pharmacological activities and molecular mechanisms, co-administration, toxicity, derivatives, and drug delivery systems in the last 5 years. The study results found that FMN has a wide range of pharmacological activities in neurological disorders, organ damage and cancer, showing great potential for clinical application and broad prospects. Researchers are exploring various types of delivery systems, including nanoparticle carriers, ligand modifications and polymer microspheres. These advanced delivery systems can enhance the stability of FMN, prolong its release time *in vivo*, and improve targeting, thereby optimizing its therapeutic efficacy and reducing side effects, and greatly improving its bioavailability. In conclusion, FMN is a natural metabolite with considerable research value, and its diverse biological activities make it a promising candidate for drug development and medical research.

## 1 Introduction

Flavonoids are a class of natural metabolites widely found in plants and foods, belonging to an important branch of polyphenolic compounds, which have a wide range of biological activities, including anti-inflammatory, antidiabetic, and anticancer (Billowria et al., 2024). Because of their different structural characteristics, they are divided into different types, thus displaying various functions. Among them, isoflavones are a unique large class of flavonoids, mainly found in soybeans and legumes, which have estrogenic activity and can be combined with estrogen receptors. Phytoestrogens are not hormones in the traditional sense, but a secondary metabolite product of plants. It is similar to animal estrogen in structure and function, and can have many effects on health, which may help to prevent certain hormone-related diseases, such as breast cancer and osteoporosis (Patra et al., 2023).

FMN is a kind of isoflavone phytoestrogen, which is widely found in plants and common diets. It has important biological activities, and has become the current research hotspot in the field of hormones. Modern research has shown that FMN is one of the active ingredients in some commonly used botanical drugs, such as *Astragalus mongholicus, Pueraria montana*, and *Trifolium pratense*. With the deepening of modern pharmacological research, the medicinal value of FMN has been further developed. Currently, the research on the pharmacological effects, combination application and drug delivery system of FMN has been carried out in-depth, indicating that FMN has a variety of pharmacological activities and clinical application potential as a single-component drug, and is expected to become a promising drug. In the past few years, the pharmacological effects of FMN have been widely reported. However, most of the previous reports were scattered and lacked systematic summarization and generalization. Therefore, this article aims to summarize the reports on FMN in the past 5 years, which will provide a basis for the future development and clinical application of FMN.

## 2 Chemical properties and plant origin of FMN

**FIGURE 1:**
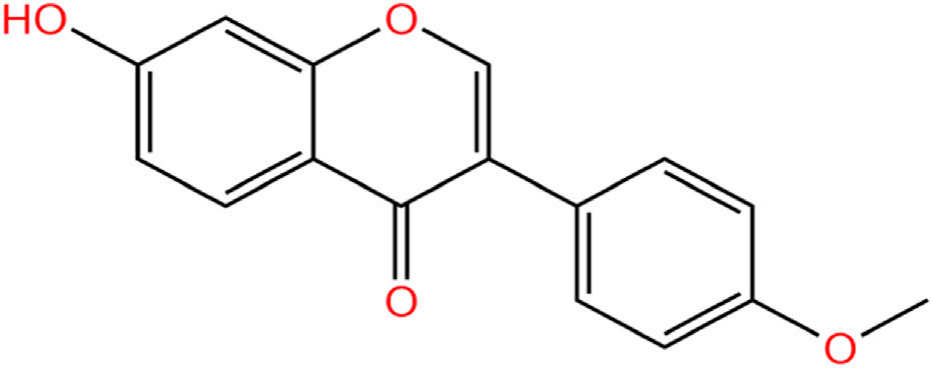
Chemical structures of FMN.

FMN is a well-known flavonoid with the parent structure of isoflavones, so it is also known as 7-hydroxy-4′-methoxy isoflavone, with the molecular formula C_16_H_12_O_4_, relative molecular weight 268.26 (Křížová et al., 2019), and the chemical structure is shown in Figure 1. Structurally, FMN has only one phenolic hydroxyl group, so it is poorly soluble in water and easily soluble in organic solvents such as methanol, ethyl acetate, and ether. FMN is a secondary metabolite produced by plants, predominantly found in legumes, as shown in Table 1. In addition, it is also present in normal diets, such as milk (Andersen et al., 2009), beer (Lapcík et al., 1998), and coffee (Alves et al., 2010).

**TABLE 1:** Plant origin of FMN.

Plant species	Family	Present site	Ref.
*Butea superba Roxb*	Leguminosae	Tuberous root	Ngamrojanavanich et al. (2007)
*Cicer arietinum* L	Leguminosae	Seed	Gao et al. (2015)
*Dalbergia tonkinensis*	Leguminosae	Leaves, Heartwood	Cuong et al. (2022)
*Glycyrrhiza glabra*	Leguminosae	Root	Vaya et al. (1997)
*Pueraria montana var. lobata* (Willd.)	Leguminosae	Root	Cui et al. (2018)
*Spatholobus suberectus*	Leguminosae	Stem	Park et al. (2017)
*Trifolium subterraneum* L.	Leguminosae	Whole plant	Sakakibara et al. (2004)
*Trifolium pratense* L.	Leguminosae	Whole plant	Sakakibara et al. (2004)
*Astragalus mongholicus* Bge.	Leguminosae	Root	Pan et al. (2021)
*Dalbergia odorifera*	Leguminosae	Heartwood	Zhao et al. (2020)
*Sophora flavescens*	Leguminosae	Root	Hwang et al. (2005)
*Sophora secundiflora*	Leguminosae	Leaves	Aly et al. (2020)
*Amorpha fruticosa* L.	Leguminosae	Leaves	Cui et al. (2017)
*Andira inermis*	Leguminosae	Stem, Leaves	Kraft et al. (2000)

## 3 Pharmacological activities and molecular mechanisms of FMN

### 3.1 Neurological diseases

#### 3.1.1 Alzheimer’s disease

Alzheimer’s disease (AD) is a progressive neurodegenerative disease and has been listed as a global priority public health problem (Ren and Qu, 2023), and its pathogenesis is related to the hyperphosphorylation of tau protein and Aβ deposition. It had been found that FMN, isolated from *Sophora secundiflora*, could improve memory problems by restoring the level of oxidative stress in brain tissues and modulating acetylcholinesterase activity. It was a potential neuroprotective agent for further research and development of AD treatment in the pharmaceutical industry (Aly et al., 2020; Sugimoto et al., 2021). Inflammatory responses could lead to vascular damage in the brain, disrupting the blood-brain barrier, promoting Aβ deposition and aggregation, and accelerating the development of AD (Silva et al., 2019). Therefore, inhibition of cerebral vascular inflammation plays a crucial role in the pathogenesis of AD. Fan et al. (2022) treated human brain microvascular endothelial cells (HBMECs) with Aβ_25–35_ for 12 h and performed quantitative real-time reverse transcription polymerase chain reaction (RT-qPCR). The results of western blot analysis showed that the expression of intracellular adhesion molecule-1 (ICAM-1), vascular cell adhesion molecule-1 (VCAM-1) and E-selectin protein increased, and nuclear factor-κB [NF-κB (p65)] was translocated to the nucleus of HBMECs. But after the administration of FMN, the expression levels of the above three decreased and NF-κB activation was inhibited, which may be related to the release of FMN blocking kelch-like ECH-associated protein-1 (Keap1) and activating the nuclear factor erythroid 2-related factor 2 (Nrf2) signaling pathway. Similarly, FMN exerted anti-neuroinflammatory effects by targeting peroxisome proliferator-activated receptor coactivator-1α (PGC-1α) and bidirectionally regulating NF-κB signaling pathway and Nrf2/Heme oxygenase-1 (HO-1) signaling pathway, thereby inhibiting tau protein hyperphosphorylation. Significantly FMN improve cognitive dysfunction in mice caused by high-fat feeding (Fu et al., 2019).

#### 3.1.2 Depression and anxiety

Neurotransmitters are chemicals in the nervous system that regulate the transmission of signals between neurons. Disruptions in their function can lead to alterations in emotional and cognitive processes, potentially contributing to conditions such as depression and anxiety (Lee, 2013). In mice models of depression established by chronic corticosterone (CORT) injection, FMN significantly increased sucrose preference and shortened the resting time of forced swimming. Using ELISA kits and Western blot analysis, researchers found that FMN decreased serum corticosterone levels and upregulated the protein expression levels of the glucocorticoid receptor (GR) and brain-derived neurotrophic factor (BDNF) in the hippocampus. Additionally, FMN mitigated CORT-induced neuronal damage in the CA1 and CA3 regions of the hippocampus and promoted neurogenesis in this brain area (Zhang et al., 2022). In addition, the inflammatory response could indirectly affect the development of depression by influencing body system diseases. Yang J. et al. (2023) demonstrated for the first time that FMN improved depressive behaviors in mice with myocardial infarction-associated depression. The underlying mechanism involves the reduction of interleukin-6 (IL-6) and IL-17A by inhibiting glycogen synthase kinase-3β (GSK-3β) activity and downregulating downstream signaling molecules, including Notch homolog 1 (Notch1) and CCAAT/enhancer binding protein α (C/EBPα). This, in turn, promoted macrophage/microglia polarization toward the M2 phenotype, reduced neuroinflammation, increased BDNF and 5-hydroxytryptamine (5-HT) levels, and mitigated the progression of depression in mice. To investigate the anxiolytic effect of FMN, Wang X. S. et al. (2019) used a complete Freund’s adjuvant (CFA)-induced anxiety mice model in mice and administered FMN continuously for 14 days. The results showed that FMN significantly increased the open-arm time in the elevated plus maze test and the central area time in the open-field experiment in mice, and had a favorable anxiolytic effect. Molecular mechanism studies showed that FMN may alleviate the inflammation and neuronal hyperexcitability in the basolateral amygdala and alleviate the anxiety-like behavior in mice by inhibiting the NF-κB and N-methyl-D-aspartate (NMDA)/cAMP-response element binding protein (CREB) signaling pathways. Thus, FMN can be used as a new candidate drug for the treatment of depression and anxiety disorders.

#### 3.1.3 Cerebral ischemia-reperfusion injury

Cerebral ischemia-reperfusion (I/R) injury refers to the phenomenon that the injury is further aggravated and deteriorates during reperfusion after reversible ischemic brain injury, which has a high mortality and disability rate. FMN could target Janus kinase 2 (JAK2)/signal transducers and activators of transcription 3 (STAT3) and phosphatidylinositol 3-kinase (PI3K)/protein kinase B (AKT)/extracellular regulated protein kinases (ERK) signaling pathways. It could significantly reduce the level of inflammatory factors, increase the number of dendritic spines in neurons, and increase the expression of βIII-tubulin, growth-associated protein 43 (GAP-43), nerve growth factor (NGF) and BDNF. FMN could protect the neurological dysfunction and pathological changes of brain tissue in rats with arterial occlusion from both anti-inflammatory and neuroprotective aspects (Wu Y. et al., 2020; Yu et al., 2022). Other studies showed that poly(ADPribose) polymerase-1 (PARP-1) and poly (ADP-ribose) glycohydrolase (PARG) played key roles in ischemic neuronal cell death and disease progression. When the activities of both are inhibited, they could effectively reduce the area of cerebral infarction, eliminate the inflammatory response, and restore neurological function in stroke patients (Cuzzocrea and Wang, 2005; Liu et al., 2022). On this basis, Luo et al. (2024) used PARP-1 inhibitor PJ34 and PARG inhibitor ethacridine lactate to investigate whether FMN’s mechanism of action involves PARP-1 and PARG. The result found that FMN significantly reduced PARP1, PARG, apoptosis-inducing factor (AIF), cysteinyl aspartate-specific protease 3 (caspase-3) and p53 protein in rats with cerebral ischemia-reperfusion injury, and increased the expression of endogenous neuroprotective gene Iduna, effectively reducing the area of cerebral infarction and neuronal apoptosis in rats. These findings suggested that FMN may serve as a potential inhibitor of PARP-1 and PARG for the treatment of cerebral I/R injury.

#### 3.1.4 Spinal cord injury

Spinal cord injury (SCI) is a devastating neurological state that leads to impaired sensory and motor functions and has a significant impact on the global healthcare system (Anjum et al., 2020). FMN can improve neurological function and exert neuroprotective effects in spinal cord injury by reducing local apoptosis and inflammatory cell infiltration. Zhou and Zhang (2023) treated PC12 cells with lipopolysaccharide (LPS) and different concentrations of FMN (50 μM, 100 μM, 200 μM). They found that FMN decreased tumor necrosis factor-α (TNF-α), IL-1β, IL-6, p-p65 NF-κB, nucleotide-binding oligomerization domain-like receptor protein 3 (NLRP3) and lactate dehydrogenase (LDH) levels, increased proliferating cell nuclear antigen (PCNA) expression, increased cell viability, inhibited apoptosis, and significantly ameliorated LPS-induced inflammatory injury in neuronal cells. In another report, Fu et al. (2023) demonstrated *in vivo* and *in vitro* that FMN attenuated microglial cell inflammatory response to spinal cord injury by inhibiting the epidermal growth factor receptor (EGFR)/mitogen-activated protein kinase (p38 MAPK) signaling pathway, thereby promoting nerve injury repair in rat spinal cord.

##### 3.1.4.1 Peripheral neuropathy

Oxaliplatin, a platinum-based chemotherapeutic agent, is commonly used in the treatment of metastatic rectal cancer but is limited in clinical application due to its severe neuropathy. Fang et al. (2020) found that FMN had a favorable ameliorative effect on oxaliplatin-induced peripheral neuropathy and did not affect the chemotherapeutic function of oxaliplatin. Molecular mechanism studies demonstrated that FMN had this effect because it could target the activation of the Nrf2 pathway, increase the activity of the phase II metabolizing enzyme glutathione S-transferase pi 1 (GSTP1), and inhibit oxaliplatin-induced peripheral neuropathy by protecting mitochondrial function.

**FIGURE 2:**
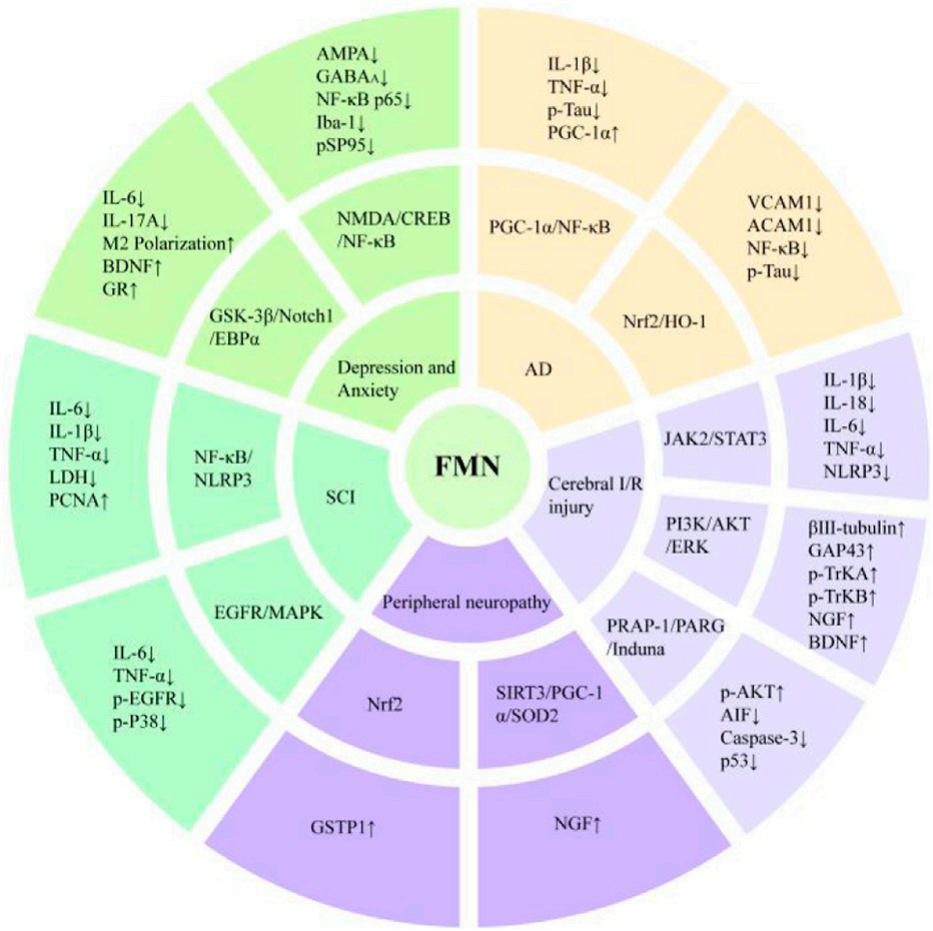
Neuroprotective mechanism of FMN.

Diabetic peripheral neuropathy is one of the most common chronic complications of diabetes. The main symptoms include spontaneous refractory pain, which may last for weeks to months. Currently, there are no drugs specifically for the treatment of diabetic peripheral neuropathy on the market. Drugs for the prevention and treatment of diabetic peripheral neuropathy also have strong side effects, which make clinical application difficult (Yang et al., 2022). In both *in vivo* and *in vitro* experiments, FMN was shown to be a potential therapeutic agent for diabetic peripheral neuropathy. It promoted the release of NGF by activating silenced information regulatory factor 3 (SIRT3), inhibited oxidative stress, enhanced neuronal survival, restored mitochondrial function, and significantly increased the pain threshold in neuropathic rats, as demonstrated in the hot-plate and tail-dip experiments (Oza and Kulkarni, 2020; Jiang et al., 2023). The neuroprotective mechanism of FMN is shown in Figure 2.

### 3.2 Organ injuries

#### 3.2.1 Lung injury

FMN has good therapeutic effects on pulmonary arterial hypertension (PAH), hyperoxia-induced acute lung injury (ALI), and chronic obstructive pulmonary disease (COPD). Cai et al. (2019) used monocrotaline (MCT) to induce PAH in rats and administered FMN. The results indicated that FMN may provide a protective effect against MCT-induced PAH in rats by inhibiting the PI3K/AKT and ERK pathways, decreasing the expression of α-smooth muscle actin (α-SMA), PCNA, and B-cell lymphoma 2 (Bcl-2), thereby preventing the excessive proliferation of pulmonary artery smooth muscle cells (PASMCs) and pulmonary vascular remodeling. Similarly, Wu J. et al. (2020) found that FMN inhibited pulmonary vascular remodeling in MCT-induced PAH rats by down-regulating the expression of transforming growth factor-β1 (TGF-β1), matrix metalloproteinase-2 (MMP-2), MMP-9, collagen types I and III, and fibronectin. It also reduced the dense focal collagen deposition induced by MCT. In addition, FMN alleviated MCT-induced lung inflammation by decreasing the expression of inflammatory cytokines, including TNF-α, IL-1β, and monocyte chemotactic protein-1 (MCP-1). Chen et al. (2021) exposed C57BL/6 mice to hyperoxia for 72 h to establish an ALI model. Intraperitoneal injection of FMN significantly reduced hyperoxia-induced increases in lung water content, pro-inflammatory cytokine levels, and lung neutrophil infiltration in mice. Western blot analysis showed that FMN increased the expression and activity of Nrf2, HO-1, and superoxide dismutase (SOD), thereby promoting the polarization of macrophages to the M2 phenotype. In another report, Li et al. (2024a) established the COPD mice model by exposing mice to cigarette smoke (CS) for 24 weeks and treated bronchial epithelial BEAS-2B cells with CS extract for 24 h to investigate the *in vivo* and *in vitro* effects of FMN on COPD. The results showed that FMN inhibited the activation of aryl hydrocarbon receptor (AhR)/CYP1A1 and AKT/mechanistic target of rapamycin (mTOR) signaling pathways, reduced CS-induced inflammatory response, endoplasmic reticulum stress and apoptosis, and effectively improved lung function and airway remodeling in COPD mice, both *in vivo* and *in vitro*.

#### 3.2.2 Liver injury

Liao et al. (2021) found that FMN may be a potential active ingredient in the prevention or treatment of liver injury by network pharmacology combined with biochemical assays, which could reduce hepatotoxicity and improve liver function through inflammatory molecular pathways. In the concanavalin A (ConA)-induced autoimmune hepatitis (AIH) mice model, FMN-treated mice showed reduced hepatocyte swelling and inflammatory cell infiltration (Liu G. et al., 2021). Further molecular mechanism studies showed that FMN inhibited the activation of the NF-κB signaling pathway and NLRP3 inflammasome, and reduced the serum levels of alanine aminotransferase (ALT) and aspartate aminotransferase (AST) in mice. At the same time, FMN upregulated the expression of Bcl-2, downregulated the expression of Bcl-2-associated X protein (Bax), cleaved caspase 9, cleaved caspase 3 expression in liver tissue, and inhibited ConA-induced apoptosis in mice hepatocytes.

The process of hepatic ischemia-reperfusion injury involves the regulation of multiple factors, among which mitochondrial damage is one of the causative factors (Cannistrà et al., 2016). Ma et al. (2022) found that FMN could protect against I/R-induced hepatic injury by restoring the function of mitochondrial autophagy. Compared with the model group, the FMN group could effectively improve sinus congestion and cell swelling in rat liver tissue, and reduce serum AST, ALT, TNF-α and IL-1β levels. In addition, through the prohibitin-2 (PHB2)/PTEN-induced putative kinase protein 1 (PINK1)/Parkin signaling pathway, the levels of LC3II, Beclin1, p62, cyclooxygenase-2 (COX2), COX4, MMP and adenosine triphosphate (ATP) were increased, and the liver mitochondrial autophagy function and energy metabolism failure were restored. In addition, during this process, the activity of antioxidant proteins glutathione (GSH), catalase (CAT), GSH-PX in the FMN treatment group recovered, and the levels of reactive oxygen species (ROS) and malondialdehyde (MDA) decreased. These findings suggest that FMN can inhibit oxidative stress in the liver and restore mitochondrial function.

Liver injury is not only a direct lesion at the liver, but other pathologies can also indirectly lead to the occurrence of liver injury, such as cholestasis. Cholestasis is characterized by intracellular bile acid (BA) overload in hepatocytes, based on which FMN can improve hepatic/systemic BA metabolism and protect the liver from liver injury caused by intracellular retention of BA. Yang et al. (2019) induced cholestasis in the mice model using α-naphthylisothiocyanate (ANIT) and used FMN by gavage for 10 days. The results showed that FMN significantly improved gallbladder enlargement and bile color change in the cholestasis model mice. qRT-PCR and Western blot analyses revealed that FMN targeted sirtuin 1 (SIRT1) and peroxisome proliferator-activated receptor alpha (PPARα), reducing the levels of inflammatory markers, promoting bile flow from hepatocytes to the bile ducts, improving liver and portal vein bile acid transport, and inhibiting ANIT-induced liver injury and cholestasis. These findings suggest that FMN may serve as a potential therapeutic strategy for cholestatic liver disease.

#### 3.2.3 Kidney injury

Kidney disease has become an significant threat to human death. There are about 850 million patients with kidney disease worldwide, and the global prevalence of chronic kidney disease (CKD) is more than 10% (Feng et al., 2023). Regulation of cell death is a necessary condition for maintaining normal physiological homeostasis. Various forms of cell death, including apoptosis, autophagy, necrosis, and pyroptosis, are involved in the pathogenesis of kidney disease. Iron ptosis is the accumulation of iron-dependent lipid peroxides, which is a new mode of cell death and is associated with acute kidney injury under a variety of stimuli, such as ischemia-reperfusion, sepsis, or toxins, and CKD (Bayır et al., 2023; Li et al., 2024). FMN increased the expression of SLC7A11, glutathione peroxidase 4 (GPX4) and Nrf2, reduced the level of 4-hydroxy-2-nonenal (4-HNE), and negatively regulated iron sagging by inhibiting the translocation of Smad3 from the cytoplasm to nucleus. At the same time, it reduced the expression of fibrosis genes including α-SMA, Col1a1 and fibronectin, and hindered iron apoptosis-related fibrosis, thereby alleviating CKD (Zhu et al., 2023). In addition, FMN also had a good repair effect on drug-induced renal injury. In the rat model of kidney injury induced by gentamycin and methotrexate, FMN prevented kidney tissue damage and renal function reduction caused by gentamycin and methotrexate administration to a large extent (Aladaileh et al., 2019; Althunibat et al., 2022). This was all related to the enhancement of Nrf2, HO-1, GSH and SOD expression, reduction of MDA, TNF-α, IL-1β, COX-2, and inducible nitric oxide synthase (iNOS) levels, and enhancement of renal antioxidant and anti-inflammatory capacity. Hao et al. (2021) demonstrated the protective effect of FMN against renal injury due to cisplatin treatment by *in vivo* and *in vitro* experiments. PPARα is a ligand-activated nuclear hormone receptor transcription factor that regulates the expression of genes related to inflammation and cellular lipid metabolism and contributes to the maintenance of renal function (Iwaki et al., 2019). FMN upregulated PPARα expression in a ligand-dependent manner to increase the levels of Nrf2, HO-1, NAD(P)H: quinone oxidoreductase 1 (NQO1), and CAT, decreased the levels of MDA, TNF-α, and IL-1β, inhibited myeloperoxidase (MPO) activity, reduced renal proximal tubular cell apoptosis, oxidative stress, and inflammatory response. Thus, FMN is a promising drug to prevent drug-induced organ damage and can be used as a first-line treatment for acute kidney injury in future studies.

Diabetic nephropathy (DN) is one of the most common and serious complications of diabetes, which is related to the increased morbidity and mortality of diabetic patients (Samsu, 2021). In a high-glucose pathological state, elevated glucose levels stimulate cells to produce excessive ROS, activating various downstream inflammatory signaling pathways. This process induces and accelerates the accumulation of renal inflammatory fibrosis markers, such as fibronectin (FN) and ICAM-1, ultimately leading to renal fibrosis characterized by glomerulosclerosis (Li Y. et al., 2022). Therefore, oxidative stress is considered to be a major pathogenic factor in diabetic nephropathy. *In vivo* and *in vitro* experiments demonstrated that FMN could reduce inflammation and fibrosis within the glomerulus by increasing sirtuin-1 (Sirt1) protein levels in renal tissues, activating the Nrf2/ARE signaling pathway, promoting the expression of downstream antioxidant enzymes, inhibiting oxidative stress, and then down-regulating the levels of FN and ICAM-1. Restoring renal function indexes in mice with DN (Zhuang et al., 2020). In addition, FMN could reduce mitochondrial superoxide production and attenuate the loss of mitochondrial membrane power by increasing the expression of PGC-1α, a downstream target of Sirt1, and Mfn2 in the kidney, and decreasing the expression of Drp1 and Fis1, thus improving mitochondrial homeostasis and restoring renal function in rats (Huang et al., 2022). Based on oxidative stress injury, Smad3 directly binds to the promoter region to activate COl-I and COl-III, which promotes extracellular matrix (ECM) synthesis, leading to fibroblast activation and enhancing the pathological process of renal fibrosis (Dan Hu et al., 2023). FMN reduced Smad3, COl1a1, and COl3a1 mRNA and protein expression, attenuated α-SMA protein overactivation, reduced collagen production and deposition, and improved renal fiber structure (Lv et al., 2020). The above studies have shown that FMN can be used as a potential drug for the treatment of diabetic nephropathy.

#### 3.2.4 Gastric ulcer

**FIGURE 3:**
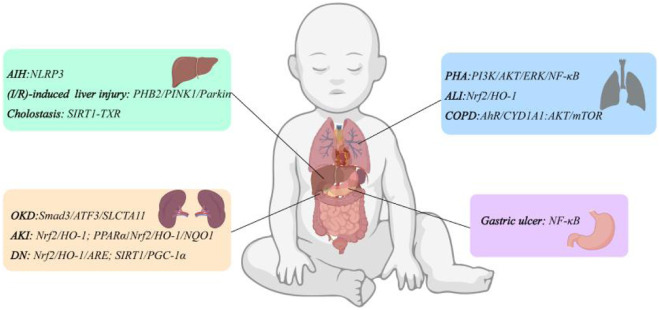
Organ protective mechanism of FMN.

Gastric ulcer is a disease characterized by chronic inflammation and barrier damage of the gastric mucosa, affecting 5%–10% of the population. Its etiology is closely related to excessive drinking, use of non-steroidal anti-inflammatory drugs and infection with *helicobacter pylori* (Mousa et al., 2019). Due to the side effects and resistance to conventional medications such as cimetidine, amoxicillin, and omeprazole, there is a need to find new therapeutic approaches. Rubab et al. (2022) used ethanol-induced gastric ulcer rats model to explore whether *Caragana ambigua* had anti-gastric ulcer effect and used liquid chromatography-mass spectrometry for component analysis. The results showed that FMN in *C. ambigua* significantly reduced the gastric ulcer index in the gastric tissue of model rats, and increased the pH value of gastric juice, gastric parietal protein and gastric mucus content. The results of histopathological observation showed that FMN could also significantly reduce gastric mucosal injury, submucosal edema and leukocyte infiltration in rats. In the rats model of gastric ulcers stimulated by non-steroidal anti-inflammatory drugs, FMN also played the same role (Mendonça et al., 2020). Yi et al. (2022) found that FMN could significantly improve the phenomenon of exfoliation necrosis and a large number of inflammatory cell infiltration in some areas of gastric mucosa induced by acetic acid in rats. At the same time, FMN upregulated the levels of CD34 protein, tight junction protein, vascular endothelial growth factor (VEGF) and nitric oxide (NO) in a dose-dependent manner, downregulated the level of human endothelin-1 (ET-1), and promoted gastric mucosal angiogenesis. In addition, compared with the model group, FMN could effectively reduce the levels of TNF-α, IL-1β, IL-6, p-P65/P65 and MPO activity, and increase the level of p-IκBα/IκBα, suggesting that FMN could play an anti-gastric ulcer role by inhibiting the activation of NF-κB signaling pathway. The above studies have shown that FMN can treat gastric ulcers through damage repair and anti-inflammatory effects. The organ protective mechanism of FMN is shown in Figure 3.

### 3.3 Cardio vascular diseases

#### 3.3.1 Atherosclerosis

Atherosclerosis (AS) is a chronic inflammatory disease characterized by lipid deposition and persistent inflammation within the arterial wall (Zhu et al., 2018). In the early stage of AS, the primary trigger of AS is vascular endothelial dysfunction, which is caused by vascular endothelial cell damage and hemodynamics (Wang et al., 2023). Zhang et al. (2021) discovered through network pharmacology and *in vitro* experiments that FMN alleviated inflammation, oxidative stress, and apoptosis responses in HUVECs induced by oxidized low-density lipoprotein by activating peroxisome proliferator-activated receptor-γ (PPAR-γ) signaling. He et al. (2024) further demonstrated that FMN upregulated the expression of α7nAChR, CD206, IL-10, SHIP1, and Arg-1, while down-regulating the expression of CD68, iNOS, COX-2, miR155-5p, IL-6, and IL-1β. FMN also promoted macrophage polarization to the M2 phenotype, thereby reducing inflammation and atherosclerosis in ApoE^−/− mice by inhibiting the JAK/STAT signaling pathway. In addition, foam cells play a key role in the development of AS, composed of macrophages, vascular smooth muscle cells, and other types of cells that adhere to endothelial cells, ingesting accumulated lipids and lipid products as foci for growing lesions (Galindo et al., 2023). Ma et al. (2020) found that FMN reduced vascular smooth muscle cell and macrophage-derived foam cell formation and their accumulation in the arterial wall, which may be associated with decreased SRA expression and reduced monocyte adhesion. Therefore, foam cells may serve as a potential breakthrough for FMN in the treatment of AS.

#### 3.3.2 Protecting blood vessels

Zhou et al. (2022) found that FMN can reduce vascular endothelial injury caused by deep vein thrombosis in rats by increasing the expression of endothelial nitric oxide synthase (eNOS) and NO production. On this basis, Wu et al. (2020) further determined through *in vivo* and *in vitro* experiments that the mechanism by which FMN exerted its effects was due to the activation of the ERK1/2 and AKT signaling pathways, which in turn significantly enhanced endothelial function. In addition, FMN dose-dependently increased the levels of insulin-like growth factor 1 (IGF-1), ICAM-1, and VEGF, and promoted the proliferation, migration, and angiogenesis of HUVECs (Liang et al., 2019).

Diabetes induces vascular endothelial dysfunction, a critical and initiating factor in the development of diabetic vascular complications (Shi and Vanhoutte, 2017). The JAK/STAT signaling pathway is involved in the development of diabetic vascular complications (Banes-Berceli et al., 2007). FMN acted as an inhibitor of JAK, significantly reducing the phosphorylation and mRNA expression of JAK2 and STAT, as well as down-regulating the protein and mRNA levels of IL-1β and ICAM-1. Additionally, FMN restored NO synthesis and phenylephrine-induced contraction and acetylcholine-induced relaxation in the aortic tissues of rats on a high-glucose diet in a dose-dependent manner (Zhou et al., 2019). These findings suggested that FMN may be a new candidate natural metabolite for preventing and treating diabetic vascular complications.

#### 3.3.3 Protect the heart

FMN was protective against both cardiac I/R injury and cardiac fibrosis. For cardiac I/R injury, FMN significantly attenuated cardiac dysfunction, infarct size, and release of cardiac markers, and inhibited the elevation of inflammatory factors TNF-α, IL-1β, and IL-6 in rats with cardiac I/R injury, primarily by inhibiting the activation of the ROS-TXNIP-NLRP3 pathway (Wang et al., 2020). Regarding cardiac fibrosis, FMN improved mitochondrial function by downregulating MOAB mRNA expression and upregulating the mRNA expression of aldehyde dehydrogenase 2 (ALDH2) and hydroxyacyl-CoA dehydrogenase (HADH). Additionally, it significantly inhibited isoproterenol-induced cardiac fibrosis and the expression of fibrosis-related proteins and genes in mice (Qian et al., 2024).

### 3.4 Joint diseases

#### 3.4.1 Osteoporosis

Osteoporosis is a common clinical pathologic bone disease, which is the result of the imbalance between the functions of osteoblasts and osteoclasts and is most common in the elderly and postmenopausal women. Estrogen levels decline in postmenopausal women, leading to disruptions in the differentiation and activation of osteoblasts, reduced synthesis and deposition of new bone, and ultimately osteoporosis. FMN belonged to isoflavones and had estrogen-like effects. It can promote osteoblast differentiation by activating the p38 MAPK/Smad/bone morphogenetic protein (BMP) signaling pathway (Soundharrajan et al., 2019). In addition, enhanced osteoclast resorption is another important mechanism in the development of osteoporosis. Under the stimulation of inflammatory factors, mouse bone marrow-derived macrophages (BMMs) can differentiate into osteoclasts, increasing osteoclast bone phagocytosis capacity and leading to increased bone loss (de Villiers, 2023). The joint inflammatory response caused by CoCrMo particles produced by the implanted prosthetic biomaterials will also induce the formation, differentiation, and maturation of osteoclasts, and ultimately transform the homeostasis of bone metabolism into osteolysis (Yin et al., 2023). In response to this process, FMN could prevent this process by inhibiting the activation of the NF-κB/MAPK signaling pathway and inhibit osteolysis triggered by osteoclast over-resorption (Yu et al., 2023), making it a potential therapeutic agent for the prevention and treatment of periprosthetic osteolysis. It can be seen that FMN can improve osteoporosis disease by reducing the inflammatory response, inhibiting BMMs differentiation to osteoclasts, and reducing osteoclast resorption, which is instructive for the treatment of osteoporosis.

#### 3.4.2 Osteoarthritis

Osteoarthritis (OA) is a degenerative joint disease characterized by cartilage degeneration and inflammatory responses (Abramoff and Caldera, 2020). Studies had shown that FMN reduces the expression of cartilage-degrading enzymes, such as MMP-1, MMP-3, and MMP-13, in primary rat chondrocytes, effectively counteracting IL-1β-induced catabolism and enhancing chondrocyte viability (Cho et al., 2019). Furthermore, FMN inhibited the inflammatory response by blocking the phosphorylation of related components in the NF-κB/MAPK signaling pathway. It also reduced the levels of cartilage catabolic markers, including MMP-3, MMP-13, and thrombospondin motifs 5 (ADAMTS5), in a concentration-dependent manner, while increasing the level of the cartilage-specific marker COL2A1 and improving bone and joint damage in OA mice (Ni et al., 2023). In addition to targeting chondrocyte degradation, FMN also reduced NO production and PGE2 expression, upregulated the levels of type II collagen and Aggrecan, and thus improved chondrocyte ECM synthesis and inhibited cartilage surface destruction and bone mineral formation in OA rats by modulating the PTEN/AKT/NF-κB signaling pathway, and this change was dose-dependent (Jia et al., 2022).

### 3.5 Skin diseases

#### 3.5.1 Atopic dermatitis

Atopic dermatitis is a recurrent, chronic, non-infectious inflammatory skin disease characterized by persistent itching. It is the largest component of non-fatal diseases worldwide and mainly occurs in infants and children (Sroka-Tomaszewska and Trzeciak, 2021). Previous studies showed that FMN inhibited the expression of intracellular murine double minute 2 (MDM2), hypoxia-inducible factor 1 alpha (HIF-1α), and NF-κB, while reducing the levels of thymic stromal lymphopoietin (TSLP) and VEGF (Han et al., 2023). This results in decreased ear swelling and thickness in mice with acute ear edema, suggesting that FMN had potential as a treatment for inflammatory skin diseases. On this basis, Yuan et al. (2021) demonstrated that FMN could effectively alleviate AD symptoms *in vivo* and *in vitro* experiments. The underlying mechanism appeared to involve the activation of the G protein-coupled estrogen receptor (GPER), which upregulated A20 protein and mRNA expression. This, in turn, affected the ubiquitination of signal transducer and activator of transcription 1 (STAT1) and STAT3, thereby inhibiting TSLP production. Thus, TSLP is identified as a key target of FMN in the treatment of AD.

#### 3.5.2 Psoriasis

Psoriasis is a common chronic inflammatory skin disease, which is mainly characterized by distinctive erythematous plaques with white scales (Kamiya et al., 2019). Currently, mild psoriasis is mainly treated by topical therapy with steroids, vitamin D analogs, calcium-modulated phosphatase inhibitors, keratolytic agents, and phototherapy, while moderate and severe psoriasis is mainly treated by systemic therapy (Calabrese et al., 2023). Although these therapies are effective in the short term, they are associated with potential side effects and contraindications, limiting their suitability for long-term use. Therefore, there is an urgent need for affordable, safe, and effective treatments. *In vitro* experiments found that FMN could significantly inhibit the growth of HaCaT cells, and 20 μM of FMN could minimize the levels of TNF-α and IL-6, which had good anti-inflammatory activity (Xu et al., 2024). This result was confirmed *in vivo* experiments, and it was found that the interferon (IFN) signaling pathway was inhibited, which could effectively reduce the expression of related inflammatory chemokines, and significantly improve the erythema, scales and thickness of skin lesions in the psoriasis mice model.

### 3.6 Diabetes

Diabetes is a metabolic disease characterized by hyperglycemia and is mainly classified as Type I and Type II, of which Type 1 diabetes mellitus (T1DM) is caused by the destruction of pancreatic β-cells, resulting in an almost complete loss of, or a severe deficiency in, insulin secretion (American Diabetes Association, 2011). Pancreatic cell damage caused by oxidative stress stimulation is one of the key factors promoting the development of T1DM. Based on this, Chen et al. (2024) demonstrated through *in vivo* and *in vitro* experiments that FMN binds to Keap1, activating the Keap1/Nrf2 signaling pathway. This promoted the translocation of Nrf2 from the cytoplasm to the nucleus, enhanced the expression of antioxidant proteins HO-1 and NQO1, and prevented excessive ROS production in pancreatic cells. As a result, FMN effectively mitigated alloxan-induced pancreatic β-cell and DNA damage, lowered blood glucose levels, and increased insulin content.

### 3.7 Lipid metabolism

Non-alcoholic fatty liver disease (NAFLD) is one of the most common liver diseases worldwide, characterized by steatosis caused by the accumulation of triglycerides in hepatocytes (Sheka et al., 2020). Non-alcoholic steatohepatitis (NASH) is a progressive form of NAFLD, which can further develop into cirrhosis and hepatocellular carcinoma with the development of time. Liao et al. (2024) established the mice model of NASH using a methionine-choline-deficient diet. Following intervention with FMN, liver function and hepatocyte steatosis were significantly improved. This effect was attributed to FMN’s ability to increase the expression and activity of SIRT1, promote the deacetylation of PGC-1α, and enhance the transcriptional activity of PPARα. These effects led to the upregulation of fatty acid oxidation (FAO) capacity, as well as increased levels of carnitine, acyl-CoA dehydrogenase medium-chain (ACADM), and carnitine palmitoyl transferase 1A (CPT1A), thus promoting liver fatty acid β-oxidation and regulating lipid metabolism. Finally, the steatosis of hepatocytes in NASH mice was improved. Furthermore, if it developed into NASH, FMN promoted the nuclear translocation of transcription factor EB (TFEB) by activating adenosine monophosphate-activated protein kinase (AMPK). This activation enhanced lysosome biogenesis and the fusion of autophagosomes with lysosomes, alleviating autophagic flux blockage and inducing lipid droplet degradation to prevent cellular lipid accumulation (Wang et al., 2019). In addition, FMN could act as a prebiotic to regulate intestinal microbial flora, thereby improving host metabolism and preventing obesity (Naudhani et al., 2021).

### 3.8 Antioxidation

Oxidative stress is a disorder of cellular function and environmental homeostasis caused by the accumulation of overproduction of ROS, which can trigger multiple cellular pathways and induce organismal damage (Tang et al., 2022). Nrf is known as the“main regulator”of the antioxidant response and is a key redox-sensitive transcription factor. When Nrf is activated, it will promote the expression of phase II detoxification enzymes and antioxidant enzymes, improve oxidative stress, promote cell survival, and maintain cellular redox homeostasis (Hybertson et al., 2011). Flap necrosis is a common postoperative complication, and hypoxia leads to secondary damage to the flap tissue. By increasing the expression of Nrf2, FMN could activate the expression of downstream target antioxidant/phase II detoxification enzymes to play a transcriptional function, thereby increasing the activity of SOD and GSH-Px. At the same time, the expression of VEGF was increased and angiogenesis was enhanced (Li et al., 2022). It could be seen that FMN exerted a strong antioxidant effect through the Nrf2-driven antioxidant defense system to restore flap tissue damage. In addition, FMN could also inhibit the sustained production of ROS through the Nrf2/HO-1 pathway, alleviate acute kidney injury due to oxidative stress and improve kidney function in rats (Aladaileh et al., 2019). The main pharmacological activity mechanism of FMN is shown in Table 2.

**TABLE 2:** The main pharmacological activity mechanism of FMN.

Disease type	Cell/Animal models	Test concentration	Signal pathway	Molecular target	Ref.
AD	HBMECs, THP-1	2, 5, 7.5 and10 μM	Nrf2/HO-1	↑Nrf2; ↓NF-κB, ICAM-1, VCAM-1, eselectin	Fan et al. (2022)
	HFD mice	20 and 40 mg/kg	PGC-1α/NF-κB	↑PGC-1α,p-GSK-3β(Ser9), Nrf2, HO-1; ↓IL-1β, TNF-α, p-NF-κB, TC, TG, Tau	Fu et al. (2019)
Depression	depressive mice	20 mg/kg	-	↑GR, BDNF; ↓CORT	Zhang et al. (2022)
	RAW264.7, BV2; depression mice	20 μM; 20 or 40 mg/kg	GSK-3β/Notch1/EBPα	↑BDNF, 5-HT, Bcl-2/Bax, IL-10, PPARγ, Arg1, Fizz1, Ym1; ↓GSK-3β, cleaved caspase-3, caspase-3, IL-6, IL-17A, iNOS, CXCL10, TNF-α, IL-1β, Notch1, C/EBPα	Yang et al. (2023a)
Anxiety	chronic inflammatory pain mice	25 mg/kg	NMDA/CREB, NF-κB	↓NMDA, AMPA, CBP, p-GluN2B-t1472, p-GluA1-s831, p-GluA1-s845, p-GluN2B-s1303, GluN2B, GluN2A, PSD95, GABAA α2, GABAA γ2, Iba-1	Wang et al. (2019a)
Cerebral I/R injury	MCAO rats	30 mg/kg	JAK2/STAT3	↓IL-18, TNF-α, IL-6, IL-1β, p-JAK2, p-STAT3, NLRP3, ASC, cl-Caspase-1, cl-IL-1β	Yu et al. (2022)
	MCAO rats	30 mg/kg	PI3K/AKT/ERK	↑β III-tubulin, GAP-43, NGF, BDNF, p-Trk A, p-Trk B, p-AKT, p-ERK 1/2	Wu et al. (2020a)
	MCAO rats	10 mg/kg	PARP-1/PARG/Iduna	↑*Iduna*, p-AKT; ↓PARP-1, PARG, Caspase-3, p53, AIF	Luo et al. (2024)
SCI	PC12	50, 100 and 200 μM	NF-κB/NLRP3	↑PCNA; ↓TNF-α, IL-1β, IL-6, p-p65 NF-κB, NLRP3, LDH	Zhou and Zhang (2023)
	Primary microglia, HMC3; SCI rats	2.5, 5, and 10 nM; 20 and 40 mg/kg	EGFR/p38 MAPK	↓TNF-α, IL-6, p-EGFR, p-p38	Fu et al. (2023)
OIPN	ND7/23, CT-26, Caco-2, DLD-1, HCT-116, PC9, A649, H1975, HCC8827, H520, BxPC3, Panc1, HUVECs, OIPN mice	0.1, 1, 10 and 25 μM; 10 mg/kg	KEAP1-Nrf2-GSTP1	↑Nrf2, GSTP1; ↓ROS	Fang et al. (2020)
Diabetic perineuropathy	type 2 diabetic neuropathy rats	10, 20 and 40 mg/kg	-	↑SIRT1, NGF, GSH, SOD, catalase; ↓cholesterol, triglyceride, insulin, MDA, ROS	Oza and Kulkarni (2020)
	RSC96	5, 10 and 25 μM	SIRT3/PGC-1α/SOD2	↑SIRT1, *Ho-1, Sod1, Sod2*, NGF, PGC-1α, *Tfam, Tfb2 m*; ↓MDA, ROS	Jiang et al. (2023)
PAH	PAH rats	10, 30 and 60 mg/kg	PI3K/AKT/ERK	↑Bax/Bcl-2, cleaved caspase-3; ↓IL-1β, IL-6, MDA, α-SMA, PCNA, p-AKT, p-ERK	Cai et al. (2019)
	PAH rats	10, 30 and 60 mg/kg	ERK/NF-κB	↓TGF-β1, MMP-2, MMP-9, p-ERK, p-NF-κB, TNF-α, IL-1β, MCP-1, type I collagen, type III collagen, FN	Wu et al. (2020b)
ALI	PMVECs; ALI mice	10, 20, 60, 80 and 160 μM; 10 and 100 mg/kg	Nrf2/HO-1	↑Nrf2, HO-1, SOD, Arg1, CD163; ↓MDA, IL-1β, IL-6, MCP1, CXCL10	Chen et al. (2021)
COPD	BEAS-2B; COPD mice	50 μM; 50 mg/kg	AhR/CYP1A1, AKT/mTOR	↑Bcl-2, CYP1A1; ↓TNF-α, CXCL1, IL-10, IL-1β, IL-8, CCL22, Bax, cleaved caspase-3, GRP78, CHOP, ATF6, p-ERK, p-EIF2α, p-AKT, p-mTOR	Li et al. (2024a)
AIH	AIH mice	50 and 100 mg/kg	NLRP3	↑Bcl-2; ↓Bax, cleaved caspase 9, cleaved caspase 3, TNF-α, IL-6, IL-1β, p-NF-κB p65, IκBα, NLRP3, ALT, AST	Liu et al. (2021a)
(I/R-) induced liver injury	I/R-induced liver injury rats	30, 60, and 90 mg/kg	PHB2/PINK1/Parkin	↑PHB2, COX2, COX4, GSH, CAT, GSH-Px, MMP, Parkin, PINK1, LC3 II, P62, Beclin1, ATP; ↓PARL, PGAM5, AST, ALT, TNF-α, IL-1β, ROS, MDA	Ma et al. (2022)
Cholestasis	HepG2; acute and chronic hepatic cholestasis mice	10, 20 and 50 mg/kg	SIRT1-FXR	↑SIRT1, BSEP, MRP2, ACOX1, OATP1, OATP4, NTCP, PPARα, CYP7A1, CYP27A1, CYP8B1, CYP7B1; ↓ALT, AST, ALP, γ-GT, TBIL, IL-6, TNF-α, IL-1β, OSTα, OSTβ, MRP3, MRP4, p-JNK	Yang et al. (2019a)
CKD	pTECs, CKD rats	20, 40 and 80 μM; 40 mg/kg	Smad3/ATF3/SLC7A11	↑SLC7A11, GSH, Nrf2, GPX4; ↓α-SMA, Col1a1, 4-HNE, KIM-1, MDA, p-Smad3, ATF3, FN	Zhu et al. (2023)
AKI	AKI rats	60 mg/kg	Nrf2/HO-1	↑GSH, SOD, CAT, Nrf2, HO-1, Bcl-2; ↓MDA, NF-κB p65, IL-1β, IL-6, TNF-α, Bax, caspase-3, creatinine, urea, protein carbonyl	Althunibat et al. (2022)
	AKI rats	10, 20 and 40 mg/kg	Nrf2/HO-1	↑GSH, SOD, GSH, ATP, CAT, Nrf2, HO-1, Bcl-2; ↓Kim-1, MDA, NF-κB p65, IL-1β, IL-6, TNF-α, Bax, caspase-3, creatinine, urea, protein carbonyl	Aladaileh et al. (2019)
	HK-2, AKI rats	10 and 25 μM; 75 mg/kg	PPARα/Nrf2/HO-1/NQO1	↑PPARα, Nrf2, HO-1, NQO1, CAT; ↓BUN, creatinine, MDA, MPO, TNF-α, IL-1β	Hao et al. (2021)
DN	GMCs, DN mice	5, 10 and 20 μM; 25 and 50 mg/kg	Nrf2/ARE	↑Sirt1, HO-1, SOD-1, Nrf2; ↓FN, ICAM-1, Keap1, ROS, FBG, TG, TC, Cr, BUN	Zhuang et al. (2020)
	HK-2, DN rats	10 and 20 μM; 20 mg/kg	Sirt1/PGC-1α	↑Sirt1, Bcl-2, Mfn2, PGC-1α; ↓Bax, cleaved-caspase-3, Drp1, Fis1, ROS	Huang et al. (2022)
	DN mice	25 and 50 mg/kg	-	↑ISI, SOD, GSH-Px, CAT; ↓FBG, FINS, IRI, TG, TC, Ucr, BUN, Scr, ACR, MDA, COL-III, α-SMA, COl1a1, COl3a1, smad3	Lv et al. (2020)
gastric ulcer	gastric ulcer rats	25, 50 and 50 mg/kg	NF-κB	↑VEGF, NO, CD34, ZO-1, p-IκBα/IκBα, occludin; ↓TNF-α, IL-1β, IL-6, MPO, ET-1, p-P65/P65	Yi et al. (2022)
AS	HUVECs	40 µM	PPAR-γ	↑SOD, eNOS, PPAR-γ; ↓TNF-α, IL-1β, COX-2, MDA, cleaved caspase-3, ROS	Zhang et al. (2021)
	AS mice	15, 30 and 60 mg/kg	JAK/STAT	↑CD206, IL-10, SHIP1, Arg-1, α7nAChR; ↓CD68, iNOS, COX-2, miR155-5p, IL-6, IL-1β, p-JAK2, p-STAT3, LDL-C, ox-LDL, TC, TG	He et al. (2024)
	AS mice	10 mg/kg	-	↑VSMCs, α-SMA, KLF4, Arg1; ↓SRA, CD68, VCAM-1, ICAM-1, PECAM-1, iNOS, ROS, TNF-α, IL-1β, IL-6	Ma et al. (2020)
Vascular injury	DVT rats	10, 20 and 40 mg/kg	-	↑p-eNOS; ↓IL-1β, IL-18, D-dimer, F1+2, TF, TM, Iκκβ, p-NF-κB p65/NF-κB p65	Zhou et al. (2022)
	HUVECs; dermal wound healing mice	10, 20 and 40 μΜ	Erk1/2/Akt	↑p-eNOS, NO, p-ERK1/2, p-AKT	Wu et al. (2020c)
	HUVECs; high-glucose diet rats	2, 20 and 200 μM; 4, 40 and 400 mg/kg	JAK/STAT	↑NO; ↓p-JAK2, p-STAT3, caspase-3, IL-6, IL-1β, ICAM-1	Zhou et al. (2019a)
I/R cardiac injury	NRCMs, MIRI rats	1 and 10 μM; 10 and 30 mg/kg	ROS-TXNIP-NLRP3	↑Bcl-2/Bax; ↓TNF-a, IL-1β, IL-6, ROS, NLRP3, LDH, AST, cTnT, INF/AAR, ROS, cleaved caspase-1, IL-1β, cleaved GSDMD, TXNIP	Wang et al. (2020a)
cardiac fibrosis	cardiac fibrosis mice	20 and 40 mg/kg	-	↑ALDH2, HADH; ↓TGF-β1, type I collagen, ROS, MOAB, α-SMA, vimentin, Col1a1, Col3a1, Acta2, Tgfb1	Qian et al. (2024)
Osteoporosis	C2C12	1.25 and 2.5 μΜ	p38MAPK/Smad/BMP	↑myogenin, myosin heavy chains, MyoD, BMP-2, BMP-7, BMP-4, ALP, RUNX2, OCN, BMPs, p38MAPK, Smad1/5/8; ↓JAK1-STAT1	Soundharrajan et al. (2019)
	BMMS; C57BL/6 J mice	10 μΜ; 10 mg/kg	NF-κB/MAPK	↓Nfatc1, c-Fos, Ctsk, TRAP, F-actin rings, NF-κB p65, IκBα	Yu et al. (2023)
OA	Primary Rat Chondrocytes	25 and 50 μΜ	-	↓MMP-13, MMP-1, MMP-3, NO, iNOS, COX-2, PGE2, CINC-2, CINC-3, fractalkine, GM-CSF, IL-1α, IL-1β, IL-4, IL-6, IL-10, LIX, MCP-1, MIP-3α, β-NGF, TIMP-1, TNF-α, VEGF	Cho et al. (2019)
	BMMs, MCPCs; C57BL/6J mice	5, 10, 20, and 40 μM	NF-κB/MAPK	↓NFATc1, c-fos, DC stamp, TRAP, ctsk, IkBα, p65, p-ERK, p-JNK, MMP-3, MMP-13, adamts5	Ni et al. (2023)
	first-passage chondrocytes, OA rats	25, 50 and 100 μM; 10 mg/kg	PTEN/AKT/NF-κB	↑type II collagen, ECM, Aggrecan; ↓NO, PGE2, COL-II, TNF-α, IL-6, iNOS, COX-2, ADAMTS5, MMP-3, MMP-13, IκBα, p65, p-AKT	Jia et al. (2022)
Atopic dermatitis	HaCaT; Atopic dermatitis mice	0.1, 1 and 10 μM; 10 mg/kg	-	↑A20, GPER; ↓TSLP	Yuan et al. (2021)
Psoriasis	HaCaT, psoriasis mice	10, 20 and 40 uM; 60 mg 2% FMN	IFN	↓IL-6, IL-17, IFN-β, IFN-γ, TNF-α, pSTAT1, p-STAT3, CD3, IRF1, Cxcl9, Cxcl10, Cxcl11, Cxcr3	Xu et al. (2024)
T1DM	MIN6; T1DM mice	3.125, 6.25 and 12.5 μM; 2.5, 5 and 10 mg/kg	Keap1/Nrf2	↑Nrf2, HO-1, NQO1; ↓ROS, Keap1, p-H2A	Chen et al. (2024)
NASH	L02; NASH mice	20, 40 and 80 μM; 25, 50 and 100 mg/kg	SIRT1/PGC-1α/PPARα	↑FAO, carnitine, ACADM, SIRT1, CPT1A; ↓IL-1β, Il-6, TNF-α, PGC-1α, TG, ALT, AST	Liao et al. (2024)
NAFLD	HepG2; NAFLD mice	10 and 20 μM; 100 mg/kg	TFEB	↑LC3B, LAMP1, ATP6V1A, AMPK, Beclin1, S6K1, PGC1α, LC3B-II, p62, TFEB, PPARα, CPT1α; ↓TG, TC, LDL-C, ALT, AST	Wang et al. (2019b)
Obesity	WSD mice	20, 60 and 100 mg/kg	-	↑occludin; ↓glucose, insulin, IL-6, IL-22, TNF-α, Muc-2, HDL, LDL	Naudhani et al. (2021)
Flap necrosis	Flap necrosis mice	25 and 50 mg/kg	PI3K/Akt/Nrf2	↑HO-1, NQO1, GCLc, GCLm, TrxR, SOD, GSH-Px, VEGF, Nrf2; ↓Keap1, TNF-α, IL-1β	Li et al. (2022b)

### 3.9 Anti-inflammation

FMN has good therapeutic effects against inflammatory diseases, such as rhinitis, mastitis, and keratitis. Huang et al. (2021) reported that FMN could inhibit IL-13-induced inflammation and mucus formation in JME/CF15 cells by activating the SIRT1/Nrf2 signaling pathway, suggesting that FMN may be a promising drug for the treatment of allergic rhinitis. Xiang et al. (2022) found that FMN could protect the breast by inhibiting the inflammatory response and enhancing the integrity of the blood-milk barrier. Specifically, FMN significantly reduced the production of inflammatory factors TNF-α and IL-1β by inhibiting the NF-kB signaling pathway and concentration-dependently increased the expression of AhR and inhibited the phosphorylation of Src, thus exerting an anti-mastitis effect. Additionally, FMN increased the expression of mammary epithelial tight junction proteins (claudin-3, occludin, and ZO-1), which enhanced the integrity of the blood-milk barrier and provided protection to the mammary gland. In the model of fungal keratitis induced by Aspergillus fumigatus in mice, researchers observed that TSLP and TSLPR were overexpressed in epithelial cells and infiltrating immune cells. After treatment with FMN, the expression of TSLP, TSLPR, and inflammatory factors was reduced, ROS production decreased, and the migration of macrophages and neutrophils was inhibited, alleviating the cornea’s inflammatory response (Feng et al., 2024). In addition, FMN enhanced the migration of HCECs from human corneal cells and promoted corneal epithelial repair. Thus, it could be seen that FMN had the potential to be an inhibitor of TSLP to treat fungal keratitis.

### 3.10 Antitumor

#### 3.10.1 Inhibition of tumor cell proliferation

##### 3.10.1.1 COX-2/cyclin

COX-2 is expressed in many types of tumor cells, which regulates cell growth and participates in tumorigenesis and carcinogenesis through mitosis (Cheng et al., 2012). There is increasing evidence suggesting that COX-2 could serve as a potential target to reduce cancer risk (Hashemi Goradel et al., 2019). The continuous program of mitosis is known as the cell cycle, which consists of the G0, G1, S, G2, and M phases. The G1 phase is an important period that affects cell division and development. It is mediated by cyclin-dependent kinases (CDKs) and regulatory cyclin subunits, which determine whether cells further self-renew and develop (Massagué, 2004). Therefore, the G1 phase of the cell cycle can be used as a key target to control the proliferation of tumor cells. Chen et al. (2023) demonstrated that FMN effectively inhibited the proliferation of KYSE170 and KYSE150 cells by significantly reducing the mRNA and protein expression levels of COX-2 and cyclin D1, while inducing G1 phase arrest. The incidence of esophageal cancer in mice treated with FMN was significantly reduced at 18 weeks (0/15 vs. 2/15) and 24 weeks (6/15 vs. 13/15). In addition, FMN also exhibited a strong anti-proliferative effect on MCF-7 and MDA-MB-468 cells, where it significantly increased the proportion of G0/G1 phase cells after treatment (Liu et al., 2024).

##### 3.10.1.2 EGFR

EGFR is a tyrosine kinase receptor that is often overexpressed or mutated in patients with non-small cell lung cancer (NSCLC), leading to uncontrolled cell proliferation and tumor formation (Riely et al., 2024). Tyrosine kinase inhibitors, such as gefitinib, erlotinib and osimertinib, have become the first-line treatment for patients with EGFR activation mutations. However, its clinical application is limited due to drug resistance. FMN was found to have the potential to become a new EGFR inhibitor and show a significant inhibitory effect on both osimertinib-sensitive and resistant non-small cell lung cancer cells, which could greatly reduce the generation of drug resistance (Yu et al., 2020). Further *in vitro* and *in vivo* studies demonstrated that FMN binds to both WT and mutant EGFR, reducing EGFR kinase activity and inhibiting downstream signaling. This, in turn, activated GSK-3β and decreased the expression of myeloid leukemia sequence 1 (Mcl-1), without causing significant toxicity to the vital organs of mice. These findings highlight the importance of developing targeted therapies for the inhibition of tumor proliferation.

#### 3.10.2 Anti-tumor angiogenesis

HIF-1 is a heterodimeric protein consisting of HIF-1α protein and HIF-1β protein, which can activate the expression of target genes involved in angiogenesis, such as VEGF (Semenza, 2003). During cancer cell proliferation, tumor cell growth is promoted by increasing VEGF expression, enhancing vascular permeability, and stimulating tumor angiogenesis (Ahmad and Nawaz, 2022). Therefore, VEGF can be used as a target to inhibit tumor proliferation. Zhang et al. (2019) established Balb/c nude mice model of cervical cancer by inoculating HeLa cells and gavaged FMN every 7 days for a month. The results showed that during the drug intervention, the mice in the FMN group had no obvious adverse reactions and were in good condition, while the mice in the cisplatin group had poor appetite, listlessness, and reduced activity. The tumor inhibition rate in the FMN group was comparable to that in the cisplatin group, and the tumor inhibition rate was 50.17%. Further analysis using RT-qPCR and western blotting showed that the expression levels of HIF-1α, VEGF mRNA, and protein in the cervical cancer tissues of mice in the FMN group were significantly lower than those in the positive control group. It could be seen that FMN inhibited the proliferation and growth of cervical cancer cells by inhibiting the expression of HIF-1-α and VEGF. In addition, FMN also had a significant inhibitory effect on the proliferation of bladder cancer cells. Zhou H. et al. (2023) used two bladder cancer cell lines, TM4 and 5,637, to explore the mechanism of action of FMN in the treatment of bladder cancer through transcriptomics. After 24, 48, and 72 h of FMN treatment, the growth of bladder cancer cell lines T24 and 5,637 was inhibited in a dose-dependent manner, without affecting the viability of non-tumor cells. Further analysis revealed that the above phenomenon was attributed to the fact that FMN mediated VEGF expression through the regulation of fibroblast growth factor receptor (FGFR) and its receptor, which in turn impaired tumor angiogenesis and inhibited cancer cell proliferation.

#### 3.10.3 Inhibition of tumor migration invasion

##### 3.10.3.1 ERK

The ERK pathway is a classical and important pathway in the MAPK signaling pathway, which transmits extracellular signals to the nucleus and regulates the expression of related genes, and is highly expressed in aggressive cancer cells (Krueger et al., 2001; Guo et al., 2022), such as nasopharyngeal carcinoma. In addition to the compounds clinically used for the treatment of nasopharyngeal carcinoma, FMN is also considered to be a therapeutically promising compound, which can achieve antiproliferative and invasive effects through effective inhibition of the oncogenic ERK1/2 pathway and the Lamin A/C signaling pathway, thereby modulating the nasopharyngeal carcinoma tumor microenvironment and inhibiting the migration and invasion of cancer cells (Ying et al., 2019).

#### 3.10.4 Inducing tumor cell apoptosis

Apoptosis is a form of programmed cell death that plays a crucial role in normal embryonic development, maintenance of homeostasis in adult tissues, and suppression of carcinogenesis (Willis et al., 2003). Mitochondrial function is closely related to cancer development, and mitochondrial dysfunction, induced by aberrant oncogenic and tumor suppressor signals, alters cellular metabolic pathways, disrupts redox homeostasis, and contributes to resistance to apoptosis (Luo et al., 2020). The Bcl-2 family of proteins, mainly located in the outer membrane of the mitochondrion, is a key factor in regulating cellular death and an upstream regulator of the caspases cascade, which can be disrupted by disrupting the inner membrane of the mitochondrion. It can effectively promote the release of pro-regulatory factors from mitochondria by disrupting the inner mitochondrial membrane and drive apoptosis (Bruckheimer et al., 1998). FMN, as a candidate anticancer drug, could release cytochrome C (cyto C) directly through the mitochondrial pathway and activate the cascade reaction of caspase-9, caspase-3 and PARP, which ultimately lead to FaDu cell death (Oh et al., 2020). In addition, both *in vivo* and *in vitro* experiments demonstrated that FMN upregulated the levels of LDH, Bax, Apaf-1, and caspase-3 in a dose-dependent manner, and downregulated the levels of ERα, p-PI3KCA^Tyr317^, p-AKT^Ser473^ (Hu et al., 2019). Based on these findings, we concluded that FMN induced osteosarcoma cell death via a mitochondria-dependent signaling pathway.

Mitochondrial autophagy is a critical cellular process and a key regulatory mechanism for the removal of damaged mitochondria, essential for maintaining cellular homeostasis (Qiu et al., 2021). In addition, more and more data suggest that mitochondrial autophagy plays an important role in the occurrence, development, and treatment of many tumors. Li et al. (2023) collected clinical samples from triple negative breast cancer (TNBC) patients and explored the effect of FMN on TNBC. The results demonstrated that FMN inhibited mitosis by inactivating the BACH1/p53 signaling pathway, promoted the release of cyto C and cysteine asparaginase, and mediated mitochondrial autophagy, resulting in mitochondrial dysfunction. In this process, FMN delivered ROS to mitochondria to release cyto C and activated caspase-3 and caspase-9 cascade reactions to induce apoptosis in MCF7 cells. These findings suggest that FMN may exert its anti-tumor function by hindering mitochondrial function in breast cancer cells.

#### 3.10.5 Regulation of microRNAs

MicroRNAs are small non-coding regulatory RNAs ranging from 17 to 25 nucleotides. In the past few years, research on microRNA-based therapies for cancer has begun to emerge. The expression of microRNAs can reflect either carcinogenic or tumor-suppressive effects, making them useful for tumor diagnosis and prognosis (Lee and Dutta, 2009). TNBC is the most refractory breast cancer type and the second leading cause of cancer-related deaths worldwide (Li S. et al., 2022). Currently, therapeutic options for TNBC are limited to surgery and chemotherapy. However, because it is a highly heterogeneous cancer with specific mutational signaling, targeted therapy is a promising option for the treatment of TNBC. FMN demonstrated targeted potential in the treatment of TNBC. By up-regulating the expression of miR-195 and miR-545, it could inhibit the expression of CDK4 and Raf-1 in the development of TNBC, thereby inhibiting the proliferation, migration and invasion of MDA-MB-231 and BT-549 cells (Wu et al., 2021). However, the experiment has not been validated by *in vivo* experiments, so it lacks the comprehensiveness and reliability of the experimental results, and the follow-up needs to be supplemented with *in vivo* experiments to verify the actual effect and safety in organisms. Li et al. (2020) found that FMN could inhibit the viability of osteosarcoma MG-63 cells and induce apoptosis. It was found by dual luciferase assay, western blot and qRT-PCR that FMN targetedly increased the expression of PTEN by reducing the expression of miR-214-3p. In addition, FMN also showed anti-gastric cancer activity (Wang and Zhao, 2021). *In vitro* experiments, FMN inhibited the proliferation of gastric cancer cells SGC-7901 and MGC-803 in a dose-dependent manner and significantly reduced cell colony formation, which was related to the decreased expression of miR-542-5p. The anti-gastric cancer potential of FMN *in vivo* was confirmed using an SGC-7901 xenograft model. Compared with the control group, the xenograft tumor volume of the FMN group was smaller, the tumor weight was lighter, and the expression of miR-542-5p was decreased. The results were consistent with *in vitro* experiments. In summary, natural metabolites combined with molecular targets have great prospects in treating cancer.

#### 3.10.6 Immune checkpoint inhibitor therapy

**FIGURE 4:**
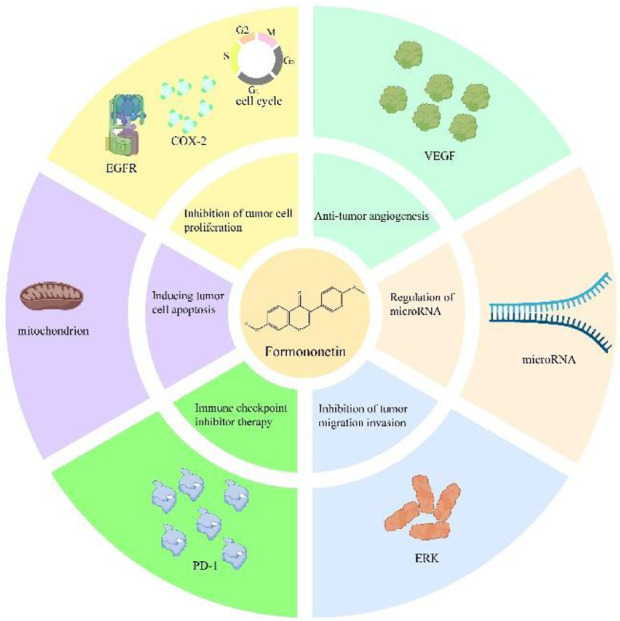
Anti-cancer mechanism of FMN.

Programmed death 1 (PD-1) is a protein expressed on T-cells. When it binds to the PD-L1 receptor on immune cells, it disrupts the effector function of lymphocytes and inhibits T-cell proliferation, thereby facilitating tumor escape from immune system attack (Safi et al., 2021). Liu et al. (2024), Wang et al. (2022) found that FMN could inhibit the occurrence of breast cancer and cervical cancer by interfering with the activation of PD-L1, but their mechanisms were different. For breast cancer, FMN mainly interfered with PD-L1 activation by inhibiting the STING-NF-κB signaling pathway. For cervical cancer, FMN mainly inhibited MYC protein expression and STAT3 activation through RAS/ERK and JAK1/STAT3 signaling pathways to inhibit PD-L1 synthesis. At the same time, it also promoted lysosomal biogenesis and induced PD-L1 degradation. Consequently, FMN could block the binding of PD-1 to PD-L1, and restore T cell activity, thereby enhancing the ability of the immune system to attack tumors. These findings suggest that FMN has the potential to serve as a PD-1/PD-L1 inhibitor for clinical use. The anti-tumor mechanism of FMN is shown in Figure 4.

### 3.11 Others

FMN has been reported to possess other pharmacological activities, such as antibacterial, antiviral, and antiallergic. Feng et al. (2024) found that FMN inhibited the growth of aspergillus fumigatus at concentrations ranging from 8–512 μg/mL, and impeded the formation of biofilm at a concentration range of 256 μg/mL. In addition, FMN also showed good inhibitory effects against *streptococcus* suis (Wang et al., 2020). Cui et al. (2021) reported that FMN had anti-cat calicivirus effect. When combined with icariin, it could play a synergistic effect. Zhou et al. (2023) suggested that FMN may be a novel anti-allergic drug with great potential to alleviate pseudoallergic reactions. In models of C48/80-stimulated BMMCs and RBL-2H3 cells, FMN inhibited immunoglobulin E-independent degranulation of dependent mast cells by reducing the release of β-hexose and histamine and the expression of inflammatory factors. Moreover, in the 2,4-dinitrobenzene-induced AD mice model, FMN reduced the local allergic reaction of AD model mice by increasing the expression of filaggrin and loricrine. Liu et al. (2021) used CKD rats and TNF-α-induced C2C12 myotubes to establish *in vivo* and *in vitro* muscle atrophy models, and found that FMN improved muscle atrophy caused by chronic renal dysfunction. This was related to FMN increasing the phosphorylation of PI3K/Akt/FoxO3 a pathway and the level of myogenic differentiation factor D (MyoD) and myogenin, thereby improving the proliferation and differentiation dysfunction of satellite cells. In the mice model of caerulein-induced pancreatitis, the researchers used FMN for continuous intragastric administration for 7 days. The results showed that FMN significantly improved pancreatic edema in mice by activating the Keap1/Nrf2 signaling pathway and reducing the levels of ROS, NLRP3 and inflammatory factors (Yang et al., 2023). In addition, FMN could also upregulate the expression of colonic tight junction proteins, restore intestinal mucosal barrier function, improve the intestinal flora environment, and provide a new solution for the treatment of pancreatitis. Park et al. (2023) believed that FMN could be used as a non-hormone treatment option for endometriosis. Through *in vivo* and *in vitro* experiments, FMN was found to achieve therapeutic effects on endometriosis by decreasing the expression of p27, p-STAT3, and p-ERK within the endometrium.

## 4 Drug combination

For many cancers, the combined treatment of drugs is superior to the effect of single treatment. This is due to the uniqueness of each drug treatment, when combined, synergistic effects can be exerted (Plana et al., 2022). The mechanisms of combination therapy for FMN are shown in Table 3.

**TABLE 3:** The mechanism of combination therapy.

FMN concentration	Combined drugs and their concentration	Treatment outcome	Cell/Animal models	Signal pathway	Molecular target	Ref.
200 μM	20 μM cisplatin	Reduce toxicity	Cochlear hair cell	PI3K/AKT/Nrf2	↑Bcl-2, Nrf2, Gclc, Gpx2, Txnrd1, HO-1, p-PI3K/PI3K, p-AKT/AKT; ↓MDA, ROS, GSSG, Bax, c-caspase-3/caspase-3	Li et al. (2024c)
40 μM; 18 mg/kg	160 µM Calycosin, 125 µM TMZ; TMZ: 30 mg/kg, Calycosin: 40 mg/kg	Synergistic effect; Increase the sensitivity of TMZ	C6; C6 xenograft mice	-	↑Bax, Cleaved Caspase-3, Cleaved Caspase-9, GFAP; ↓Bcl-2, MMP-2, MMP-9	Ni et al. (2019)
40 μM; 12.6 mg/kg	160 µM Calycosin, 125 µM TMZ; TMZ: 21 mg/kg, Calycosin: 28 mg/kg	Synergistic effect; Increase the sensitivity of TMZ	C6; C6 xenograft mice	-	↓NOS2, TNF-α, GFAP, ribitol, glutamic acid, aspartic acid, phosphonic acid, serine, ribose, methionine	Li et al. (2022d)
30 μM	20 μM Sulforaphane	Synergistic effect; PI3K/AKT/mTOR inhibitors	HeLa	PI3K/AKT/mTOR	↑ROS; ↓cyclin-D1, PCNA, CDK6, p-PI3K, p-AKT, p-mTOR	Jiang et al. (2024)
-	Cantharidin, Isofraxidin	Synergistic effect	Hep 3B2.1–7, Li-7; zebrafish	EGFR/PI3K/AKT	↓EGFR, p-PI3K/PI3K, p-AKT/AKT, BIRC5, FEN1, EGFR	Lu et al. (2024)
150 *μ*mol/L; 50 mg/kg	100 nmol/L everolimus; everolimus: 2 mg/kg	Synergistic effect	MDA-MB-468; MDA-MB-468 xenograft mice	mTOR	↑PTEN, p-4EBP-1; ↓p-mTOR, p-P70S6K, p-Akt	Zhou et al. (2019b)
40 μM or 80 μM	150 μM MET	Synergistic effect	MCF-7	ERK1/2	↓Bcl-2, p-ERK1/2	Xin et al. (2019)

### 4.1 Combination with chemotherapeutic agents

Cisplatin is a common chemotherapeutic drug in the clinic, and ototoxicity is an unavoidable side effect during its use, which can damage the inner ear and impair both hearing and balance functions in patients (Wang et al., 2023). Currently, no effective treatment exists to repair the hair cell damage induced by cisplatin. Based on this, Li et al. (2024) used cisplatin to culture cochlear hair cells *in vitro* and treated them with FMN, and found that FMN effectively reduced the accumulation of ROS and mitochondrial damage in hair cells by activating the PI3K/AKT-Nrf2 signaling pathway, restored the balance of GSH/GSSG. Therefore, FMN was a potential therapeutic agent for cisplatin-induced ototoxicity.

Glioma is a common malignant tumor of the brain, and temozolomide (TMZ) is currently the first-line drug used in clinical practice for postoperative or non-surgical chemotherapy of gliomas, which can easily penetrate the lesion site through the blood-brain barrier, but the acquired and intrinsic resistance of TMZ limits its application (Xu et al., 2020). In addition to using FMN alone, the combination of FMN and mullein isoflavones may enhance the antitumor effects of TMZ. *In vivo* and *in vitro* experiments showed that the IC50 of TMZ was significantly reduced from 1000 μM to 125 μM when the low effective concentration of calycosin (160 μM) and FMN (40 μM) were combined with TMZ. The combination of the three greatly improved the sensitivity of TMZ, significantly inhibited the growth and migration of C6 cells, and the toxicity of the drugs to the heart, liver, spleen, lung and kidney of mice was small (Ni et al., 2019). In addition, based on metabolomics and molecular biology studies, nitric oxide synthase 2 (NOS2) was a key target for FMN and calycosin to regulate TMZ in the treatment of glioma. FMN and calycosin reduced the secretion of TNF-α in the tumor area by inhibiting the expression of NOS2, and regulated amino acid metabolism, especially glutamic acid, ornithine, aspartic acid, proline and arginine, thereby increasing the cytotoxicity of TMZ on malignant glioma, inhibiting tumor growth and infiltration in C6 glioma rats, and reducing cell aggregation and GFAP expression in C6 glioma area (Li et al., 2022). The above studies showed that cancer cells acquire drug resistance because multi-gene abnormalities in tumor cells allow them to evade the effects of these drugs when patients receive treatment for a single pathway. Therefore, combination therapy had better control effects on multiple targets and a lower risk of drug resistance, which had great application prospects for treating cancer.

### 4.2 Combination with natural medicines

The PI3K/Akt/mTOR signaling pathway plays a critical role in the apoptosis and proliferation of various cancer cells. The activation of Akt/mTOR can initiate a signaling cascade that regulates apoptosis, survival, growth and proliferation, thus promoting tumorigenesis (Li et al., 2017). The combination of FMN (30 μM) and sulforaphane (20 μM) exhibited a significant synergistic effect (CI = 0.57), and inhibited the expression of cyclin-D1, PCNA and CDK6 in HeLa cells by down-regulating the PI3K/Akt/mTOR signaling pathway, reducing the proportion of cells in G0/G1 phase, hindering the entry of cells into S phase, and leading to the death of HeLa cells (Jiang et al., 2024). In addition, the combination of the two could promote the generation of ROS in HeLa cells, leading to oxidative damage, which was also one of the causes of HeLa cell death. It could be seen that the combination of FMN and sulforaphane could be used as an inhibitor of PI3K/AKT/mTOR, which had important clinical significance in the treatment of cervical cancer. Aidi injection is a widely used Chinese patent medicine for the treatment of hepatocellular carcinoma. Lu et al. (2024) used Hep3B2.1-7, Li-7 cells and zebrafish to explore the anti-hepatocarcinoma mechanism of Aidi injection. The results showed that the strong anti-hepatocarcinoma effect of Aidi injection was the result of the combined treatment of cantharidin, FMN and isofraxidin. Meta-analysis showed that BIRC5, FEN1 and EGFR could be the key targets of Aidi injection against hepatocellular carcinoma, and the three chemical components exerted synergistic inhibitory effects through their targets, respectively, which revealed the mechanism of anti-hepatocellular carcinoma of Aidi injection, and also provided a typical example for the pharmacological evaluation of other proprietary Chinese medicines.

### 4.3 Combination with targeted drugs

In another report, FMN could enhance the anti-tumor effect of everolimus on MDA-MB-468 cells, increasing the apoptosis rate by 27.9%. There was no significant loss of appetite in mice with the MDA-MB-468 cell xenograft model, and the tumor volume was reduced to half (Zhou et al., 2019). It could be seen that FMN combined with everolimus can safely inhibit tumor growth. RT-qPCR and western blot analysis revealed that both FMN and everolimus inhibited the expression of p-mTOR and p-P70s6k and increased the expression of PTEN and p-4EBP-1. However, FMN alone inhibited p-Akt expression, whereas everolimus had no inhibitory effect on this. When Akt siRNA was used to silence Akt, everolimus did not affect the expression of mTOR, and FMN still reversed the expression of p-4EBP-1 and p-P70s6k. It could be seen that the synergistic effect of FMN on everolimus plays a role by inhibiting Akt activity and inhibiting the mTOR signaling pathway. In addition, Xin et al. (2019) also found that FMN-assisted metformin (MET) in the treatment of breast cancer, which had great application prospects. The combination of 40 μM FMN and 150 μM MET or the combination of 80 μM FMN and 150 μM MET could reduce the proliferation rate of MCF-7 cells. Compared with the monotherapy, the levels of Bcl-2 and p-ERK1/2 were greatly reduced, and the apoptosis rate was increased.

## 5 Toxicity of FMN

FMN had been reported to be cytotoxic to human-immortalized epidermal cells HaCaT with an IC50 of 40.64 μM (Xu et al., 2024). Yu et al. (2023) treated BMMs with FMN at concentrations up to 20 μM without any significant cytotoxicity. However, Ni et al. (2023) further found that FMN had a greater effect on the viability of BMMs when the concentration reached 40 μM or higher, with an IC50 of 29.61 μM. Cho et al. (2019) evaluated the effect of FMN on the viability of rat primary chondrocytes using the MTT assay, and the results showed that, even at a concentration of 100 μM, FMN did not affect rat primary chondrocyte viability. In addition, FMN had no toxic effect on the growth of normal ovarian cell lines (Zhang et al., 2018). In another report, Pingale and Gupta (2023) carried out acute toxicity tests and subacute toxicity tests of FMN by intraperitoneal injection in mice. The results showed that the acute dose of FMN was 300 mg/kg, the LD50 was 103.6 mg/kg, and the NOAEL was 50 mg/kg. During the subacute toxicity experiment, the body weight, food intake, water intake and behavior of the animals did not change, and the organs did not have any toxic effects and pathological damage. Therefore, it was proved that FMN was safe and non-toxic and could be used for pharmacological and therapeutic purposes. In summary, FMN may be a relatively safe natural compound component. However, there is still a lack of subchronic toxicity, genotoxicity and reproductive toxicity experiments, and the follow-up should be expanded to carry out clinical trials of FMN.

## 6 Derivatives

FMN has low water solubility, which limits its application in the pharmaceutical industry. To solve the water solubility problem of FMN, Zhao et al. (2021) succinylated FMN using *Bacillus* amyloliquefaciens FJ18 to form the compound FMN-7-O-β-D (6″-O-succinyl)-D-glucoside (FMP), which compared to FMN, the water solubility was increased more than 106-fold. Moreover, in the mice model of isoprenaline-induced acute ischemia, the compound significantly attenuated cell membrane damage during myocardial ischemia by scavenging free radicals in serum, increasing CAT and SOD activities, and decreasing lactate dehydrogenase (LDH) activity. In another study, Yan et al. (2024) prepared seven derivatives of FMN from FMN. Among them, 9-butyl-3-(4-methoxyphenyl)-9,10-dihydro-4H,8H-chromeno[8,7-e][1,3]oxazin-4-one(2) and 9-(furan-3-ylmethyl)-3-(4-methoxyphenyl)-9,10-dihydro-4H,8H-chromeno-[8,7-e][1,3]oxazin-4-one possessed the ability to promote bone formation and inhibit osteoclastogenesis, which was superior to the effect of ipliflavone, suggesting that this compound could be a patentable candidate for anti-osteoporosis. In addition, Zuo et al. (2022) used structural modification to replace the methoxy group at position 40 of FMN with the phenoxy group to obtain a new compound that greatly improved the antihypertensive effect of FMN. Kim et al. (2019) utilized microbial transformation technology to synthesize a novel compound, FMN 7-O-phosphate. Compared with FMN, FMP showed lower cytotoxicity in RAW264.7 cells and significantly improved cell viability. At the same time, it inhibited the production of pro-inflammatory factors in a dose-dependent manner and decreased the mRNA expression of inducible iNOS and COX-2, which could be used as a promising candidate compound for new anti-inflammatory drugs.

**FIGURE 5:**
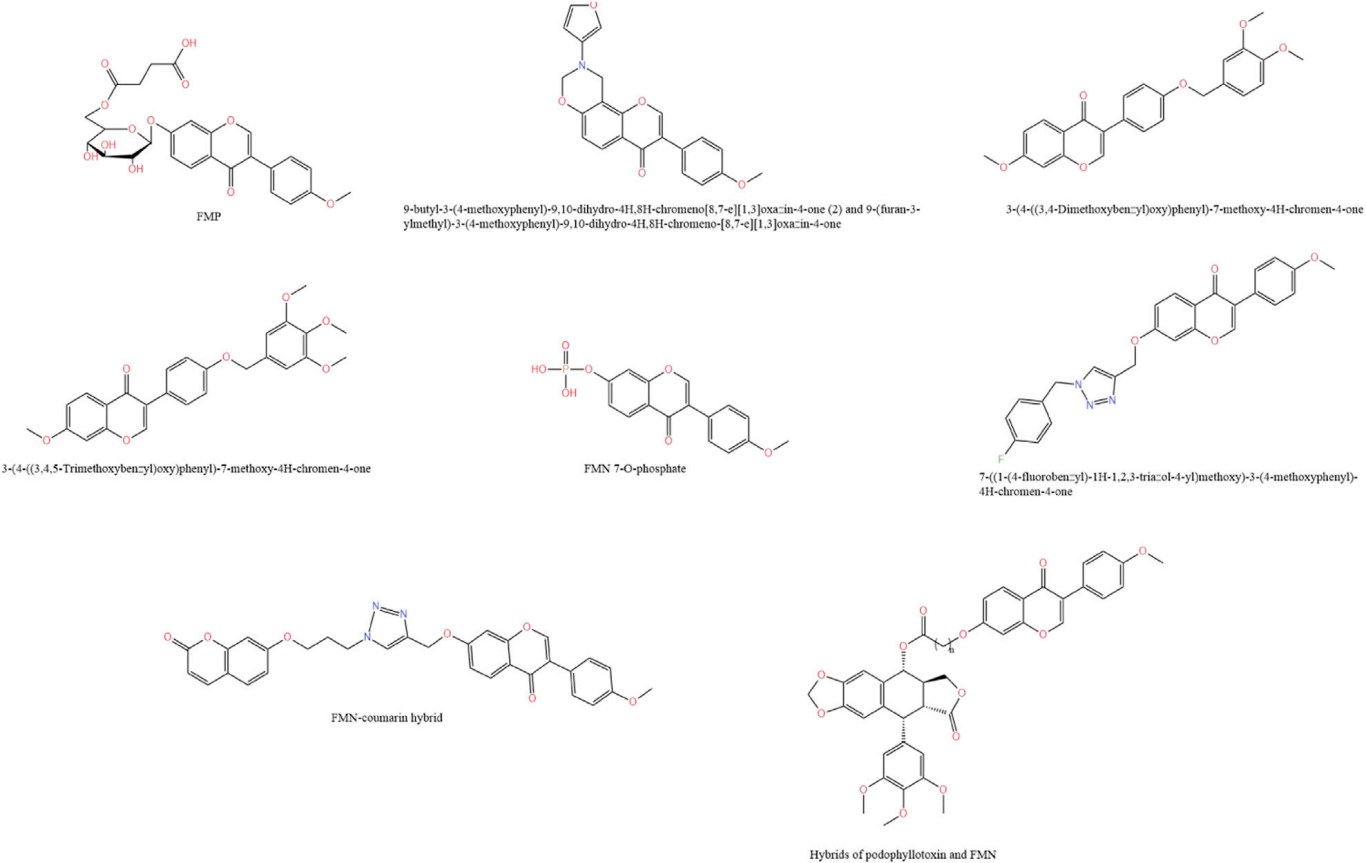
Chemical structure of FMN derivatives.

FMN can act as a Bax agonist to exert anti-cancer effects. To improve its bioavailability, Jia et al. (2023) modified its structure and obtained a new compound 7-((1-(4-fluorobenzyl)-1H-1,2,3-triazol-4-yl)methoxy)-3-(4-methoxyphenyl)-4H-chromen-4-one. The compound could significantly inhibit the growth of the A549 cell line, downregulate the expression of Bcl-2 in cells, upregulate the expression of Bax, and promote apoptosis. Its efficacy was 40 times and 6.94 times greater than that of FMN and adriamycin, respectively, indicating that the derivative had strong potential as an anti-cancer lead compound. Based on the natural FMN, Yao et al. (2019) designed and synthesized a FMN-coumarin hybrid product by molecular hybridization strategy. Compared with FMN, the hybrid product could significantly inhibit the growth and migration of gastric cancer SGC7901 cells through the wnt/β-Catenin and AKT/mTOR pathways, and could be used as an inhibitor of SIRT1 to inhibit its expression, thereby inducing cancer cell death. *In vivo*, the hybrid effectively inhibited the growth of SGC7901 xenograft tumors in nude mice without significantly affecting their body weight. Similarly, Yang et al. (2019) inserted the FMN fragment into the C4 hydroxyl position of podophyllotoxin to form a new hybrid product, which had a stronger ability to resist A549 tumor growth than podophyllotoxin. This was because FMN could be inserted into the αβ-interface to form hydrogen bonds, destroy the cytoskeleton of tubulin, and enhance anti-tumor ability. The chemical structure of FMN derivatives is shown in Figure 5.

## 7 Drug delivery systems

**FIGURE 6:**
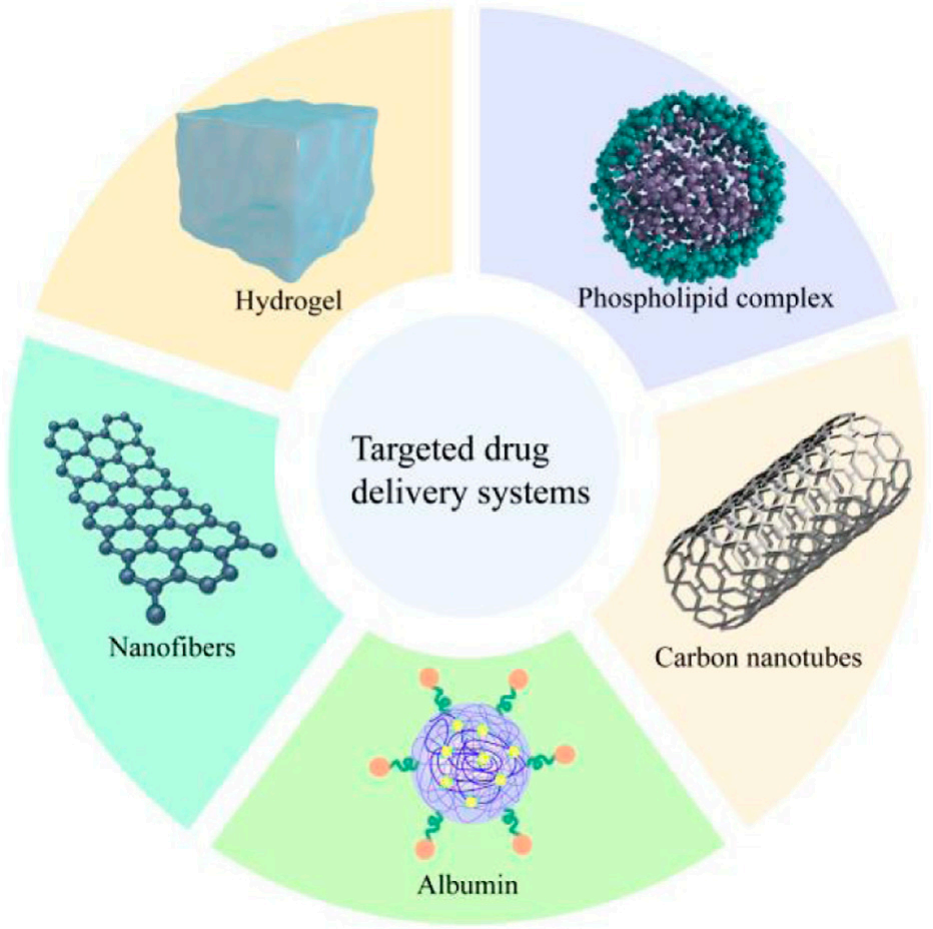
FMN’s delivery system.

Drug delivery system is the use of carriers or technologies to effectively and safely deliver drugs to specific targets in the body, which has the characteristics of optimizing the therapeutic effect of drugs, high solubility and long retention time in biological systems (Prakash, 2023). Since FMN is characterized by low water solubility and low bioavailability, limiting its application in various areas. Therefore, research based on nano-delivery systems has emerged in recent years. The following text should be added: The FMN delivery systems is shown in Figure 6.

### 7.1 Carbon nanotubes

Carbon nanotubes (CNs) are small, flexible, robust, inert, and electrically conductive materials that have been investigated for treating spinal cord injuries due to their electrical properties and nano-size. However, CNs have low hydrophilicity and poor dispersibility, which tend to lead to aggregation, so it is usually necessary to functionalize the CNs to avoid its aggregation (Imani et al., 2016). de Vasconcelos et al. (2020) used FMN to functionalize CNs, which not only improved the stability of CNs, but also further stimulated neuronal growth and cell membrane fusion through FMN, and even reduced inflammatory response. On this basis, in order to enhance the bio-interface with nerve cells, gelatin methacryloyl hydrogel was used as a scaffold, which could retain the nanocomposite of multi-walled carbon nanotubes and FMN at the inflammation site, and was directly used for the repair of neural tissues in the diseased site.

### 7.2 Protein nanoparticles

Ouyang et al. (2023) used bovine serum albumin (BSA) as raw material, and nanoprecipitation with 20:1 as the optimal ratio of albumin to FMN to prepare FMN-containing nanoparticles FMN@BSA. The nanoparticles improved the bioavailability of FMN by promoting the preferential accumulation of albumin’s affinity for SPARC protein overexpressed in lung inflammation and pulmonary fibrosis, and alleviated bleomycin-induced lung inflammation and interstitial deposition in mice by blocking NLRP3 inflammasome to slow down the pyroptosis process of macrophages. It could be seen that the nanoparticles could be used as a macrophage pyroptosis inhibitor. Compared with the commonly used first-line therapeutic drug pirfenidone, FMN@BSA was a natural product as the active ingredient, which had lower toxicity and stronger clinical transferability.

### 7.3 Cyclodextrin inclusion complexes and phospholipid complexes

It has been previously shown that β-cyclodextrin can improve the solubility of FMN (Bhardwaj and Purohit, 2023). On this basis, Wang et al. (2021), in order to improve the oral bioavailability of FMN, prepared instantly soluble nanofibers containing FMN/methyl-β-cyclodextrin (FMN/Me-β-CD) inclusion complex, which increased the solubility of FMN by about 50-fold in 20 mM Me-β-Cd aqueous solution. After that, polyvinyl alcohol nanofiber webs containing FMN/Me-β-CD inclusion complex was prepared by electrostatic spinning method, which could be dissolved in artificial saliva in about 2 s. The rapid dissolution of FMN in artificial saliva was greatly improved, which would be beneficial to the application of FMN in oral diseases. In addition, Agarwal et al. (2024) had also made efforts to improve the oral bioavailability of FMN. The researchers used phospholipid (PL) as a carrier to prepare FMN phospholipid complex (FNT-PC) using the solvent evaporation method and characterized it. The results showed that FNT-PC exhibited amorphous geometry, and the water solubility was 4 times higher than FMN. In addition, *in vivo* pharmacokinetic studies in rats by gavage showed that FNT-pc also reduced first-pass metabolism, increased gastric mucosal permeability, and significantly improved oral bioavailability.

### 7.4 Dual ligand-modified

As mentioned earlier, FMN has excellent antitumor activity, and to further enhance its clinical application value and therapeutic effect, the use of the nano-delivery system to effectively improve the bioavailability and targeting of FMN is a promising strategy. It had been reported that some researchers used epidermal growth factor receptor-targeting peptide (GE11)-modified nanoparticles (GE-NPs) loaded with paclitaxel and hyaluronic acid (HA)-modified nanoparticles (HA-NPs) encapsulated with FMN, and self-assembled both of them to construct binary nanoparticles modified by GE11 and HA dual ligands (HAGE-DTX/FMN-NPs). The binary nanoparticles showed a stronger inhibitory effect on PC3 cells with a cellular uptake rate of 59.6% compared with the single ligand-modified nanoparticles and free drug. PC3 cells were injected into the right side of Balb/c nude mice to prepare cancer xenografts by subcutaneous injection and treated by tail vein injection, and it was found that HAGE-DTX/FMN-NPs were highly aggregated at the tumor site, whereas the single ligand-modified nanoparticles and the free drug had more distribution of the drug in the liver and kidneys, which showed that the binary nanoparticles modified with dual ligands have better targeting and bioavailability and can avoid unwanted side effects (Dong et al., 2022).

### 7.5 Microspheres

Cao et al. (2023) used microfluidic technology to prepare loaded FMN microspheres, which could encapsulate and release the drug in a controlled manner. *In vitro* experiments showed that the microspheres inhibited the proliferation, migration, and apoptosis of A549 cells better than FMN, and had good anti-tumor selectivity and low toxicity to normal cells. In addition, the microspheres could induce intracellular ROS production, leading to oxidative damage and better antitumor effects.

## 8 Conclusion and future perspectives

In recent years, more and more attention has been paid to developing natural metabolite, mainly because they are derived from plants with low toxicity and high efficiency. FMN is one of the important discoveries of novel natural metabolite with considerable medicinal value. It is widely found in plants and daily diets, and is also detected in some chinese patent medicines, such as Qiliqiangxin capsule (Zhang et al., 2018), Zhenqi Fuzheng capsules (Liu et al., 2015), Guanjiekang (Wu et al., 2016) and Naoxintong capsule. This paper reviews the source, chemical properties, pharmacological activities and molecular mechanisms, co-administration, toxicity, derivatives and drug delivery system of FMN, which can provide a basis for in-depth research and clinical application of FMN.

FMN is involved in various disease processes, such as neurological disorders, organ damage and cancer, by regulating various factors, enzyme activities and signaling pathways, and it is evident that FMN has multiple pharmacodynamic effects. However, the current study still has certain flaws and limitations. First, studies on FMN have mainly focused on cellular and animal models, and the multiple pharmacological mechanisms have not been fully clarified. Although the treatment of the nervous system, organ injury and cancer is outstanding, it is still in the preliminary stage, without in-depth study, and lacks large-scale and long-term clinical trials to verify its safety and effectiveness. And the lack of FMN metabolic process, can not fully understand the drug circulation in the body. Therefore, in the future, in addition to the use of traditional *in vivo* and *in vitro* experimental analysis, other research methods should be strengthened, such as metabolomics, proteomics and genomics analysis, in order to better study the absorption, distribution and metabolic process of FMN, and how to act on the target to play a therapeutic effect. Through these studies, we can better understand the regulatory role and overall effect of FMN at the cellular molecular level. Second, to realize the maximum potential of its clinical application, further exploration of its quantitative relationship, side effects, and toxicity is needed, and this information is critical for assessing its safety and feasibility in clinical application. Finally, Although toxicity studies have been conducted on FMN, most of the tests have been at the cellular level, and more comprehensive toxicity tests such as subchronic toxicity, genotoxicity and reproductive toxicity are lacking. It is recommended to use different animal models to validate the toxicity results and extend the experimental period to obtain more data. Finally, systematic clinical trials, including randomized controlled trials, long-term efficacy observation and adverse reaction evaluation, are needed to fully understand the actual effect and safety of FMN in different diseases.

As previously mentioned, FMN has the characteristics of poor water solubility, low bioavailability and insufficient targeting. To solve those problems, structural modification and nano-delivery systems can be used as a promising solution. Numerous scholars have utilized nano-delivery systems to modulate the particle size, surface properties and carrier materials of drugs, which can significantly improve the bioavailability and targeting of FMN, and show good application prospects. At present, although studies have confirmed that the nano-delivery system has a significant improvement on the shortcomings of FMN, there is a lack of long-term safety and toxicological evaluation, and further research is needed in the future to ensure its reliability and safety in clinical applications. Meanwhile, the design of the delivery system should be further optimized to achieve higher drug loading capacity and more precise targeted release. In addition to the use of drug delivery systems alone, FMN can be combined with other treatments, such as immunotherapy, to enhance the therapeutic effect and improve the prognosis of patients; gene editing technology can be used to solve the disease directly from the root cause by precisely modifying the disease-causing genes. In the future, with the gradual enrichment of knowledge, the pharmacological activity of FMN and its molecular mechanism will be further elucidated, and the study of its structural modification and delivery system will be further optimized and innovated, which will have a new impact on the future pharmaceutical industry.
